# Chronic Hypoxia Reduced the Growth and Muscle Quality in Turbot, *Scophthalmus maximus*

**DOI:** 10.3390/ani16060861

**Published:** 2026-03-10

**Authors:** Zhongmin Guo, Yuexing Zhang, Yuliang Wei, Chenchen Bian, Mengqing Liang, Zhenyu Du, Houguo Xu, Qiang Ma

**Affiliations:** 1 National Engineering Research Center for Marine Aquaculture, Zhejiang Ocean University, Zhoushan 316022, China; 2 State Key Laboratory of Mariculture Biobreeding and Sustainable Goods, Yellow Sea Fisheries Research Institute, Chinese Academy of Fishery Sciences, Qingdao 266071, China; 3 Laboratory of Aquaculture Nutrition and Environmental Health, School of Life Sciences, East China Normal University, Shanghai 200241, China

**Keywords:** hypoxia stress, flesh quality, energy metabolism, fatty acid, amino acid, hif

## Abstract

Hypoxia is a common environmental stress in nature and aquaculture, but the adaptation mechanisms of flatfish to chronic hypoxia and its effects on flesh quality remain unclear. In this study, the turbot was cultured at control normoxia (CON, 6.5 ± 0.5 mg/L) or chronic hypoxia (CHO, 3.5 ± 0.5 mg/L) for 8 weeks; then, the growth, energy metabolism, meat quality, and the expression of related genes were measured. The CHO group significantly reduced the digestibility (*p* < 0.05), weight gain (*p* < 0.001), and body indexes (*p* < 0.01), but increased feed conversion ratio (*p* < 0.001) in turbot. Meanwhile, the CHO group decreased muscle texture, total amino acid, soluble protein (*p* < 0.001), glycogen contents, and myofiber numbers (*p* < 0.001), while increasing myofiber diameters and lactate content (*p* < 0.01). In addition, chronic hypoxia increased the hepatic angiogenesis by activating the *hif1α/vegfa* pathway (*p* < 0.05) and decreased the whole fish lipid content and liver n-3 polyunsaturated fatty acid levels (*p* < 0.05). In summary, chronic hypoxia reduced the growth, nutrient content, and flesh quality of turbot. This study provides important references for elucidating the adaptation mechanisms of flatfish to chronic hypoxia and for developing mitigation strategies.

## Simple Summary

Flatfish are highly intolerant to hypoxia stress; thus, clarifying the adaptation mechanisms of flatfish to chronic hypoxia is of great significance for healthy aquaculture development. In this study, the turbot (*Scophthalmus maximus*) was reared under either normoxia or chronic hypoxia conditions for 8 weeks. The results suggested that chronic hypoxia significantly decreased growth, body indexes, feed efficiency, whole fish lipid content, and liver n-3 polyunsaturated fatty acid levels in the turbot. In addition, chronic hypoxia decreased meat hardness, springiness, and chewiness by reducing the muscle’s total amino acid, soluble protein, glycogen contents, and myofiber numbers. These results illustrated the mechanisms of adaptation to chronic hypoxia in flatfish.

## 1. Introduction

Oxygen acts as the terminal electron acceptor in the oxidative phosphorylation process to produce adenosine triphosphate (ATP) within mitochondria, representing the foundation of life activities for aerobic organisms [1]. Unlike terrestrial animals that respire atmospheric air, aquatic animals absorb dissolved oxygen (DO) from water through gills. The DO level in water varies frequently with environmental changes, so aquatic animals are susceptible to hypoxia stress. Hypoxia of water environment is typically defined as DO level below 2 mg/L, although different fish species exhibit varying sensitivities to oxygen deprivation [2]. Over the past 50 years, global oceanic dissolved oxygen in the ocean have reduced by more than 2% (4.8  ±  2.1 petamoles) because of human activities, including the extensive use of fossil fuels and the discharge of nutrients into coastal waters [3]. In coastal waters particularly, the extent of areas experiencing dissolved oxygen level below 2 mg/L has been increasing rapidly, causing widespread effects on marine ecosystems and estuarine fisheries [4]. Under natural conditions, there are two primary drivers for chronic hypoxia in water. First, eutrophication is the major trigger of hypoxia in oceans and lakes, where excessive terrestrial nutrient inputs stimulates massive proliferation of phytoplankton [5]. Phytoplankton consume oxygen via nocturnal respiration, and when phytoplankton die and are decomposed by microorganisms in the bottom water, this process consumes large amounts of dissolved oxygen. Second, water column stratification is also a key physical condition for the formation and maintenance of bottom hypoxia. In summer, increased surface water temperature generates a significant temperature and density gradient relative to colder bottom water, hindering vertical convective exchange [6]. Oxygen produced by photosynthesis in the surface layer cannot effectively replenish the bottom layer, whereas organic matter decomposition and biological respiration at the bottom continuously consume oxygen, eventually leading to the formation of hypoxic “dead zones”, such as the Black Sea and the northern Gulf of Mexico [7]. Furthermore, global warming and ocean current changes intensify both the strength and duration of stratification-induced hypoxia [8]. In aquaculture, high stocking density is the most common reason for chronic hypoxia in aquatic animals. In turn, chronic hypoxia induced by high stocking density can reduce the survival rate, growth performance, feed efficiency, and physiological health [9,10]. In addition, failure of oxygen supply, water exchange equipment, or power supply can result in mass mortality of fish or shellfish, leading to substantial economic losses. Therefore, clarifying the adaptation mechanisms of fish to chronic hypoxia is of great significance for the sustainable development of aquaculture.

Many studies have explored the effects of acute hypoxia on oxidative stress, Hif signaling pathway, and anaerobic glycolysis in fish. Experiments in rainbow trout (*Oncorhynchus mykiss*), crucian carp (*Carassius carassius*), and largemouth bass (*Micropterus salmoides*) have confirmed that acute hypoxia can increase the lipid peroxide level, antioxidant enzyme activities (superoxide dismutase (SOD) and catalase (CAT)), and the expression of genes related to injury, as well as hif1α, glycolysis/gluconeogenesis, and angiogenesis [11,12,13]. Therefore, acute hypoxia induces oxidative stress and tissue damages by excessive production of reactive oxygen species (ROS) and imbalance of the antioxidant defense system. Meanwhile, acute hypoxia activates anaerobic glycolysis, angiogenesis and erythropoiesis via the *hif1α* signaling pathway to adapt the hypoxic environment. In addition to the shared oxidative stress and *hif1α* signaling pathway observed under acute hypoxia, research on chronic hypoxia in fish primarily focused on growth, feed efficiency, immune response, and reproductive performance. Specifically, chronic hypoxia can significantly inhibit the feed intake, growth performance, and lactate level of largemouth bass and channel catfish (*Ictalurus punctatus*), but increase the feed conversion ratio, inflammatory responses, and antioxidant enzyme activities, as well as cortisol and glycogen contents [14,15]. For the post-smolt Atlantic salmon (*Salmo salar*), chronic hypoxia up-regulated immune-related indicators and the expression of anti-bacterial genes in the head kidney [16]. In Atlantic croaker (*Micropogonias undulatus*) and common carp (*Cyprinus carpio*), chronic hypoxia inhibited gonadal development and reproductive output [17,18]. Collectively, chronic hypoxia suppresses growth, feed intake, reproductive performance, and anaerobic glycolysis, while increasing feed conversion ratio and inflammatory response. Nonetheless, the impacts of chronic hypoxia on energy metabolism, the expression of three *hifα* isoforms, muscle texture, fatty acid, and amino acid compositions remain unclear in flatfish.

The turbot is a typical cold-water benthic fish naturally distributed along the eastern Atlantic coast of Europe, ranging from southern Scandinavia to northern Africa, which is an important commercially cultured fish species in Europe [19]. Characterized by a flat body, high filet yield, tender flesh, and high nutritional value, turbot is highly favored by consumers worldwide. In China, the annual production of turbot reaches approximately 50,000 tons, primarily cultivated in the land-based recirculating system with high stocking density [20]. Unlike European turbot farming, most Chinese farms currently lack liquid-oxygen supplementation equipment, resulting in dissolved oxygen levels below 4 mg/L during high-density turbot cultivation. Our previous study has shown that turbot is highly intolerant to hypoxia, exhibiting stress responses when DO falls below 4 mg/L and mortality when it declines below 2 mg/L [20]. However, the effects of chronic hypoxia induced by high-density culture or ocean warming on the growth, physiological metabolism, and flesh quality of turbot remain unclear. This study provides insights into the adaptive mechanisms of marine fish to chronic hypoxia and offers guidance for enhancing the hypoxia tolerance of turbot.

## 2. Materials and Methods

### 2.1. Culture of Fish and Experimental Diet

Juvenile female turbot was purchased from Huanghai Aquaculture Co. Ltd. (Yantai, China). Before the formal experiment, all fish were cultured temporarily at an indoor seawater system with 4 m^3^ volume for 2 weeks and fed with the same commercial feed. The commercial feed contains 51% crude protein, 10% crude fat, less than 6% crude fiber, and less than 16% ash content. A total of 120 healthy fish (initial weight 76 ± 0.5 g) were randomly selected and divided into 2 treatment groups, and each group had three replicate tanks (20 fish/tank). The water DO level of control normoxia group (CON) was kept at 6.5 ± 0.5 mg/L, and the DO level of the chronic hypoxia group (CHO) was kept at 3.5 ± 0.5 mg/L. Low DO level in the water was maintained by reducing the airflow volume from the adjustable air pump, with dissolved oxygen levels measured six times daily using a dissolved oxygen meter (SMART SENSOR, Hongkong, China). The experimental fish were fed twice daily at 7:00 and 19:00, and the feeding amount was 3–4% of the total weight of the fish in each tank. The experiment period was 8 weeks. Throughout the experimental period, the water temperature, salinity, total ammonia nitrogen, and pH were maintained at 20.5 ± 1 °C, 26.5 ± 2.5‰, <0.02 mg/L, and 7.5 ± 0.5, respectively. These indicators were measured every 3 days, and the detection frequency was increased according to the conditions of the fish and the weather. The composition of the experimental diet is presented in Table 1.

### 2.2. Sample Collection

At the end of the culture period, the experimental fish were starved for 12 h. All fish of each tank were collectively weighed and counted to determine tank biomass. Eighteen fish were randomly selected from each group and anesthetized with MS222 (10 mg/L, Beijing Green Hengxing Biotechnology Co., Ltd., Beijing, China) for 3–5 min, and twelve fish were used for the determination of body indices and whole-body proximate composition. The remaining six fish were anesthetized, and blood samples were collected from the caudal vein. The collected blood was placed at 4 °C for 3 h, then centrifuged at 3500 rpm for 10 min. The upper layer of serum was taken in 1.5 mL centrifuge tubes. Concurrently, samples from the liver and muscle were dissected and immediately snap-frozen in liquid nitrogen for subsequent analysis.

### 2.3. Muscular Texture and Histology Analysis

Muscle samples with a size of approximately 1.5 × 1.5 × 1.0 cm were collected from the region above the left lateral line of each fish (six fish per treatment) for the assessment of muscle texture properties. Muscle texture was assayed with a texture analyzer (TMS-Pro, FTC, Sterling, VA, USA) fitted with a 25 N gravity sensor. The texture measurement was conducted under 23 °C using an 8 mm circular probe adopted, with a compression speed of 30 mm/min, and a deformation degree of 30%. For histological analysis, dorsal muscle tissues were sectioned into 0.5 cm^3^ cubes and immersed in a specialized muscle GD fixative (G1111, Wuhan Servicebio Technology Co., Ltd. Wuhan, China) for 24 h at 25 °C. After fixation, the muscle samples were processed for paraffin embedding, and serial sections of 5 μm thickness were prepared and subjected to hematoxylin-eosin (HE) staining (Wuhan Servicebio Technology Co., Ltd. Wuhan, China). The stained sections were subsequently imaged under a light microscope (Nikon DSRI2, Tokyo, Japan). All the slices were analyzed using ImageJ 1.53c (Wayne Rasband National Institutes of Health, Bethesda, MD, USA) for the statistical analysis.

### 2.4. Proximate Compositions Measure of Feed and Whole Fish

In accordance with the official protocols of the Association of Official Analytical Chemists (AOAC) [21], the moisture content was assayed via oven-drying at 105 °C for 24 h. Crude lipid content was quantified through the Soxhlet extraction method (Foss-Tecator, Hoganas, Sweden) using petroleum ether as the extraction solvent. For the determination of crude protein content, the Kjeldahl method was employed with a nitrogen-to-protein conversion factor of 6.25 (N × 6.25). Following sample digestion in a digestion furnace, crude protein levels were further measured using a Kjeldahl nitrogen analyzer (Foss, Hilleroed, Denmark). Ash content was determined at 550 °C for 4 h in a muffle furnace.

### 2.5. Analysis of Biochemical Indicators

The concentrations of glucose (F006-1-1) in the serum, glycogen (A043-1-1) in the liver and muscle tissues, as well as pyruvate (A081-1-1), lactate (A019-3-1), triglyceride (A110-2-1), total cholesterol (A111-2-1), and total protein (A045-2) in the serum, liver, and muscle tissues were assayed using commercial kits supplied by Nanjing Jiancheng Bioengineering Institute (Nanjing, China). The optical density (OD) values were measured with an enzyme microplate reader (Tecan Infinite M200, Männedorf, Switzerland). Both the specific operational procedures and absorbance detection during the experiment were conducted in strict compliance with the kit manufacturer’s instructions (available at http://www.njjcbio.com, accessed on 1 August 2024). The hemoglobin content of the blood was measured by a portable hemoglobin analyzer with electrochemistry method (Taiwan BeneCheck, Taipei City, China).

### 2.6. Analysis of Fatty Acid and Amino Acid Compositions

The fatty acid composition was determined using a gas chromatograph (GC2010Pro, Shimadzu, Kyoto, Japan). Liver samples of 50 mg were sequentially esterified with 1 mL KOH-methanol (0.5 mol/L) and boron trifluoride-methanol (14%) in a 75 °C water bath for 30 min, then extracted with n-hexane for 6 h. A silica capillary column (SH-RT-2560, 100 m × 0.25 mm × 0.20 μm, Shimadzu, Kyoto, Japan) and a flame ionization detector were used to analysis. The column temperature program was set as follows: initial temperature 100 °C; raised to 190 °C at a rate of 10 °C/min; then increased from 190 °C to 200 °C at 0.3 °C/min; finally elevated from 200 °C to 230 °C at 4 °C/min. The temperatures of the injector and detector were both maintained at 230 °C, and the flow rate of the carrier gas (hydrogen) was 3 mL/min. The results were expressed as the percentage of each fatty acid relative to the total fatty acids (% TFA). The amino acid content was analyzed using an automatic amino acid analyzer (L-8900, Hitachi, Tokyo, Japan). Weighed 20 mg of freeze-dried muscle into a 20 mL tube, then added 15 mL of 6 mol/L HCl solution, and acidified at 110 °C for 24 h with nitrogen protection. The solution was diluted to 50 mL, and a 0.5 mL aliquot was transferred to a new tube, then dried by nitrogen at 40 °C. Subsequently, 0.5 mL of 0.02 mol/L HCl solution was added to the tube for dissolution; after filtration, the sample was subjected to instrumental measurement [22].

### 2.7. Total RNA Extraction and qPCR

Liver or muscle (50–100 mg) were placed in 1.5 mL sterile centrifuge tubes, and total RNA was isolated using the RNAiso Plus reagent (Takara Bio, Dalian, China). RNA concentration and purity were assessed with a Titertek Berthold Colibri spectrophotometer (Colibri, Berlin, Germany). The A260/A280 absorbance ratio fell within 1.8–2.0, confirming that the RNA quality was good. First-strand complementary DNA (cDNA) was generated with a reverse transcription kit with gDNA eraser (Tiangen, Beijing, China). Primers for reference genes (rpl19 and β-actin) and target genes for quantitative real-time polymerase chain reaction (qPCR) were designed based on the turbot genome available on the NCBI website and synthesized by Beijing Tsingke Biotechnology Co., Ltd. (Qingdao, China). Primer details are provided in Table 2. The qPCR reaction mixture (10 μL total volume) contained 5 μL of 2× SYBR Green master mix, 1 μL of cDNA (20 ng), 0.6 μL of primers, and 3.4 μL of nuclease-free sterile water. The 96-well plates were loaded into a Roche LightCycler96 real-time PCR instrument (Roche, Basel, Switzerland) and subjected to the following protocol: denaturation at 95 °C for 30 s, followed by 40 cycles of 94 °C for 5 s and 60 °C for 30 s. Each well had two technical replicates. A melting curve analysis was subsequently performed to verify PCR product specificity. Primer amplification efficiency was determined using the formula E = 10^(−1/slope)^−1^, which yielded values between 90% and 110% in this experiment. Relative target gene expression levels were calculated using the 2^^(−ΔΔCt)^ method [23].

### 2.8. Calculation of Growth Performance and Shape Indexes

The growth and shape indexes are calculated as follows:

Survival rate (%) = final fish number/initial fish number × 100;

Weight gain (WG, %) = (final weight − initial weight)/initial weight × 100;

Feed conversion ratio (FCR) = feed intake/(final fish weight − initial fish weight);

Condition factor (K, g/cm^3^) = body weight/(body length^3^) × 100;

Hepatosomatic index (HIS, %) = (liver weight/body weight) × 100;

Viscerosomatic index (VSI, %) = (visceral weight/body weight) × 100.

### 2.9. Data Statistics and Analysis

All data were subjected to normal distribution and homogeneity of variance by One-sample Kolmogorov–Smirnov and Levene’s tests, and independent samples T-test was performed using SPSS 19.0 software for Windows (IBM corporation, Armonk, NY, USA). The FDR (Benjamini–Hochberg) method was conducted to correct the original *p*-values using the formula: adjusted *p*-value = original *p*-value × (total numbers of test/rank of this *p*-value). Significant differences were set at *p* <0.05 among the CON and CHO groups. “*”, “**”, and “***” mean significant differences (*p* < 0.05), (*p* < 0.01), and (*p* < 0.001) respectively. All results are presented as mean ± standard error of the mean (SEM), and the graphs were generated using GraphPad Prism 8.0 software.

## 3. Results

### 3.1. Effects of Chronic Hypoxia on the Growth Performance and Body Indexes of Turbot

**Figure 1:**
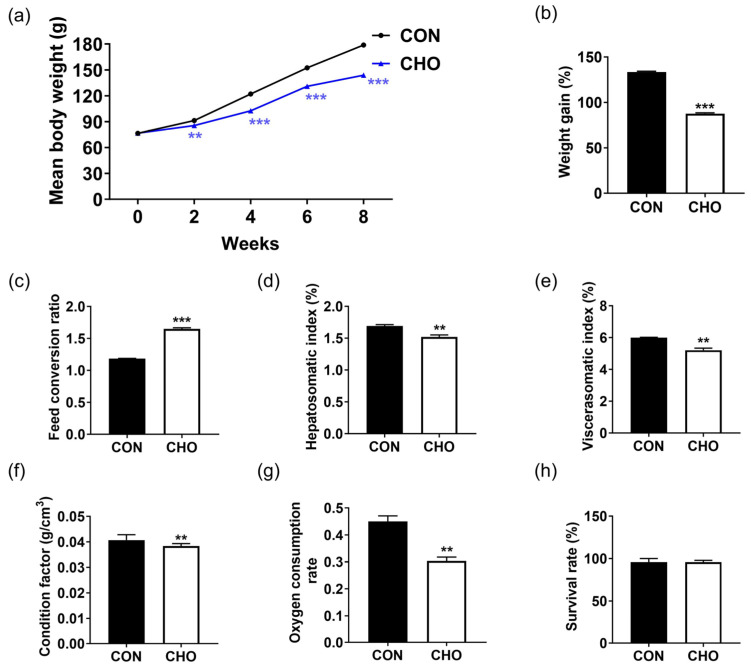
Effects of chronic hypoxia on the growth performance and body indexes of turbot. (**a**) Mean body weight; (**b**) weight gain; (**c**) feed conversion ratio; (**d**) hepatosomatic index; (**e**) viscerasomatic index; (**f**) condition factor; (**g**) oxygen consumption rate; (**h**) survival rate. Note: CON: control normoxia group; CHO: chronic hypoxia group. “**” and “***” mean significant differences (*p* < 0.01), and (*p* < 0.001) respectively, between CON and CHO groups.

Chronic hypoxia for 8 weeks significantly reduced the mean body weight, weight gain, hepatosomatic index, viscerasomatic index, condition factor, and oxygen consumption rate in the turbot (Figure 1a,b,d–g). Meanwhile, the feed conversion ratio was significantly increased in the CHO group than the CON group (Figure 1c), and chronic hypoxia had no significant effect on the survival rate of turbot (Figure 1h). These results suggest that chronic hypoxia would reduce the growth and body indexes of turbot.

### 3.2. Effects of Chronic Hypoxia on Proximate Compositions of Whole Fish, Muscle and Feces of Turbot

**Figure 2:**
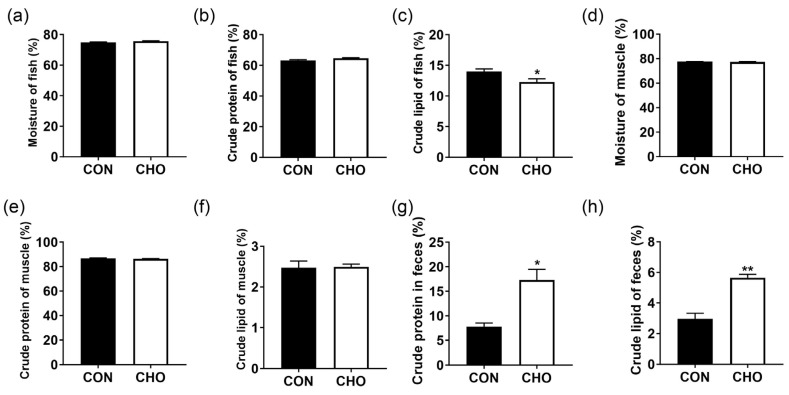
Effects of chronic hypoxia on proximate compositions of whole fish, muscle and feces of turbot. (**a**) Whole fish moisture content; (**b**) whole fish crude protein content; (**c**) whole fish crude lipid content; (**d**) muscle moisture content; (**e**) muscle crude protein content; (**f**) muscle crude lipid content; (**g**) feces crude protein content; (**h**) feces crude lipid content. Note: CON: control normoxia group; CHO: chronic hypoxia group. “*” and “**” mean significant differences (*p* < 0.05), and (*p* < 0.01) respectively, between CON and CHO groups.

As shown in Figure 2, chronic hypoxia had no significant effect on the moisture and crude protein contents in the whole fish (Figure 2a,b) and muscle (Figure 2d,e), and the crude lipid level in the muscle (Figure 2f). Compared with the CON, the lipid content of the whole fish was significantly decreased (Figure 2c) and the crude protein and crude lipid contents in the feces were significantly increased in the CHO group (Figure 2g,h). All these indicate that chronic hypoxia reduced the digestion and absorption of nutrients and crude lipid content of the whole body.

### 3.3. Effects of Chronic Hypoxia on Muscle Texture and Section of Turbot

**Figure 3:**
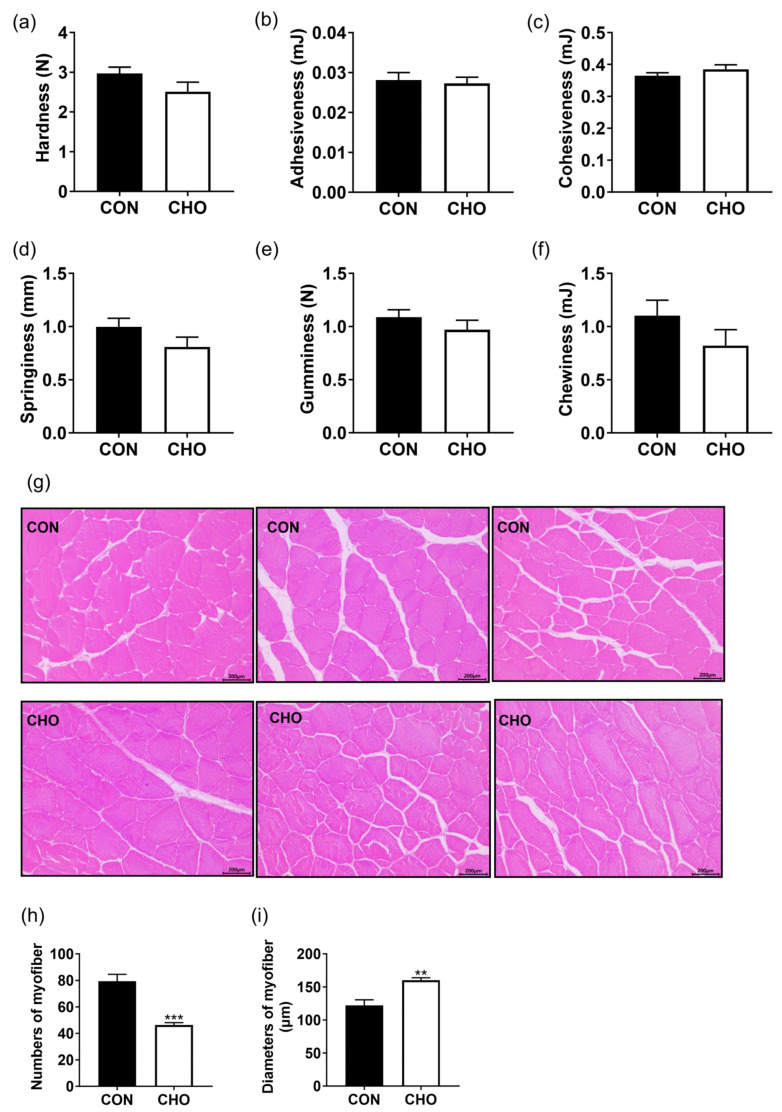
Effects of chronic hypoxia on muscle texture and section of turbot. (**a**) Hardness; (**b**) adhesiveness; (**c**) cohesiveness; (**d**) springiness; (**e**) gumminess; (**f**) chewiness; (**g**) muscle sections; (**h**) numbers of muscle myofiber; (**i**) diameters of muscle myofiber. Note: CON: control normoxia group; CHO: chronic hypoxia group. “**” and “***” mean significant differences (*p* < 0.01), and (*p* < 0.001) respectively, between CON and CHO groups.

The CHO group reduced muscle hardness, springiness, and chewiness (Figure 3a,d,f), but did not affect the adhesiveness, cohesiveness, and gumminess (Figure 3b,c,e) as much as the CON group. As shown in Figure 3g, chronic hypoxia significantly reduced the numbers of myofiber (Figure 3h), and significantly increased the diameters of myofiber (Figure 3i). These data prove that chronic hypoxia inhibited the myofiber development and muscle hardness.

### 3.4. Effects of Chronic Hypoxia on Protein Metabolism of Turbot

**Figure 4:**
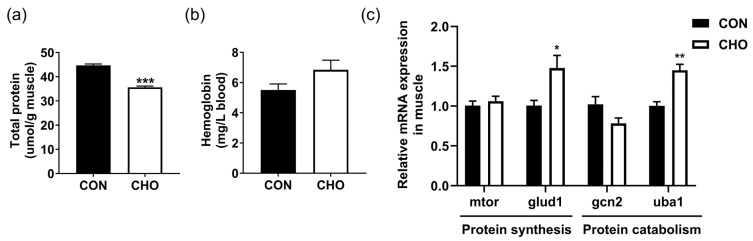
Effects of chronic hypoxia on protein metabolism of turbot. (**a**) Total muscle soluble protein content; (**b**) blood hemoglobin content; (**c**) muscle protein metabolism-related gene expression. Note: CON: control normoxia group; CHO: chronic hypoxia group. “*”, “**”, and “***” mean significant differences (*p* < 0.05), (*p* < 0.01), and (*p* < 0.001) respectively, between CON and CHO groups.

The total soluble protein content in the muscle was significantly reduced in the CHO group than the CON group (Figure 4a), while the blood hemoglobin content was increased in the CHO group (Figure 4b). Compared with the CON group, the expression of protein synthesis-related gene *glud1* and protein catabolism-related gene *uba1* were both up-regulated in the CHO group, whereas chronic hypoxia had no significant effect on expression of *mtor* and *gcn2* genes in the muscle (Figure 4c). These data suggest that chronic hypoxia disturbed protein metabolism and reduced the soluble protein content of muscle.

### 3.5. Effects of Chronic Hypoxia on the Muscle Amino Acid Compositions of Turbot

As shown in Table 3, both muscle essential and non-essential amino acid contents showed a slight decreasing trend in the CHO group than the CON group, but there was no significant difference. Above data suggest that chronic hypoxia decreased the essential and non-essential amino acid contents of muscle.

### 3.6. Effects of Chronic Hypoxia on Glucose Metabolism of Turbot

**Figure 5:**
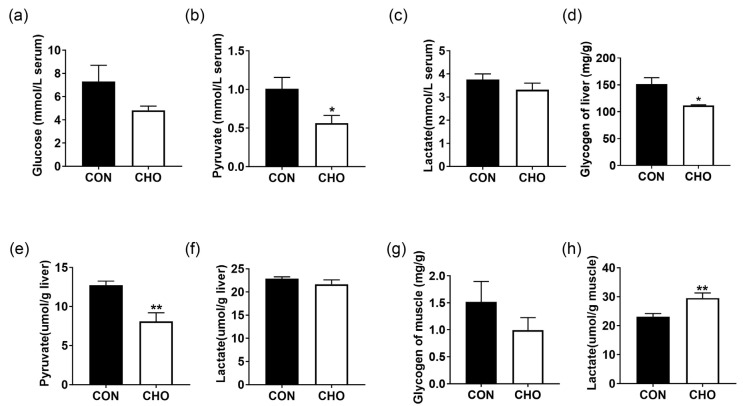
Effects of chronic hypoxia on glucose metabolism of turbot. (**a**) Serum glucose content; (**b**) serum pyruvate content; (**c**) serum lactate content; (**d**) liver glycogen content; (**e**) liver pyruvate content; (**f**) liver lactate content; (**g**) muscle glycogen content; (**h**) muscle lactate content. Note: CON: control normoxia group; CHO: chronic hypoxia group. “*” and “**” mean significant differences (*p* < 0.05), and (*p* < 0.01) respectively, between CON and CHO groups.

The glucose and pyruvate contents in the serum (Figure 5a,b), the glycogen and pyruvate contents in the liver (Figure 5d,e), as well as the glycogen content in the muscle (Figure 5g) were lower in the CHO group than the CON group. Chronic hypoxia did not affect the serum and liver lactate content (Figure 5c,f), but significantly increased the muscle lactate content (Figure 5h). These results show that chronic hypoxia could lead to the conversion of glycogen to lactate in the muscle.

### 3.7. Effects of Chronic Hypoxia on Hif, Insulin and Glycolysis Signaling Pathways

**Figure 6:**
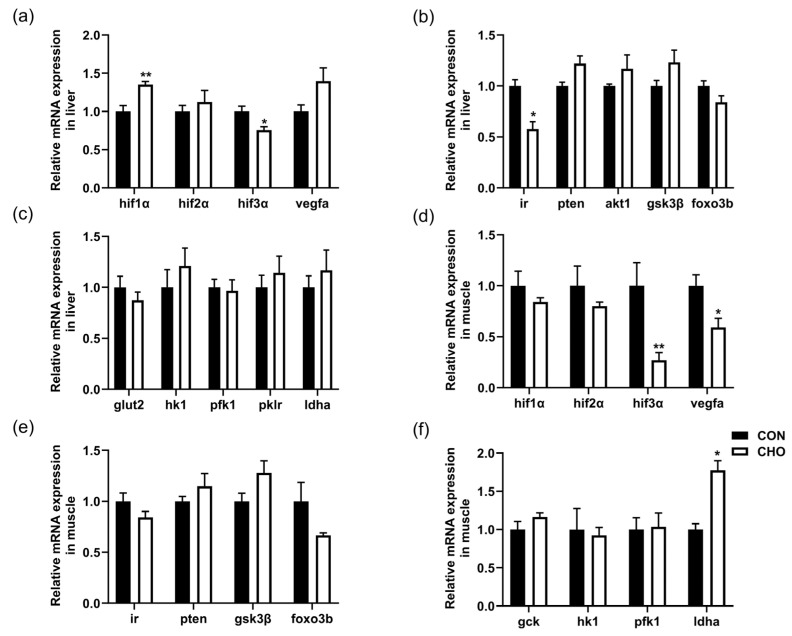
Effects of chronic hypoxia on the hif, insulin and glycolysis signaling pathways. (**a**) Liver hypoxia-inducible factor gene expression; (**b**) liver insulin signaling pathway gene expression; (**c**) liver glycolysis-related gene expression; (**d**) muscle hypoxia-inducible factor gene expression; (**e**) muscle insulin signaling pathway gene expression; (**f**) muscle glycolysis-related gene expression. Note: CON: control normoxia group; CHO: chronic hypoxia group. “*” and “**” mean significant differences (*p* < 0.05), and (*p* < 0.01) respectively, between CON and CHO groups.

As shown in Figure 6, the hepatic expression of *hif1α* and *vegfa* genes were up-regulated, but the expression of *hif3α* and *ir* genes were significantly down-regulated in the CHO group compared with the CON group (Figure 6a,b). In the muscle, the expression of *hif3α* and *vegfa* genes were down-regulated, but the gene expression of *ldha* was up-regulated in the CHO group than the CON group (Figure 6d,f). In addition, there were no significant differences on the expression of *hif2α*, insulin pathway-related genes (*pten, akt1, gsk3β,* and *foxo3b*), glycolysis pathway-related genes (*glut2, hk1, pfk1, pklr,* and *ldha*) in the liver, *hif1α*, *hif2α*, insulin pathway-related genes (*ir, pten, gsk3β,* and *foxo3b*), and glycolysis pathway-related genes (*gck, hk1,* and *pfk1*) in the muscle between the CON and CHO groups. All these findings demonstrate that chronic hypoxia promoted the hepatic angiogenesis and muscular glycolysis.

### 3.8. Effects of Chronic Hypoxia on Lipid Metabolism of Turbot

**Figure 7:**
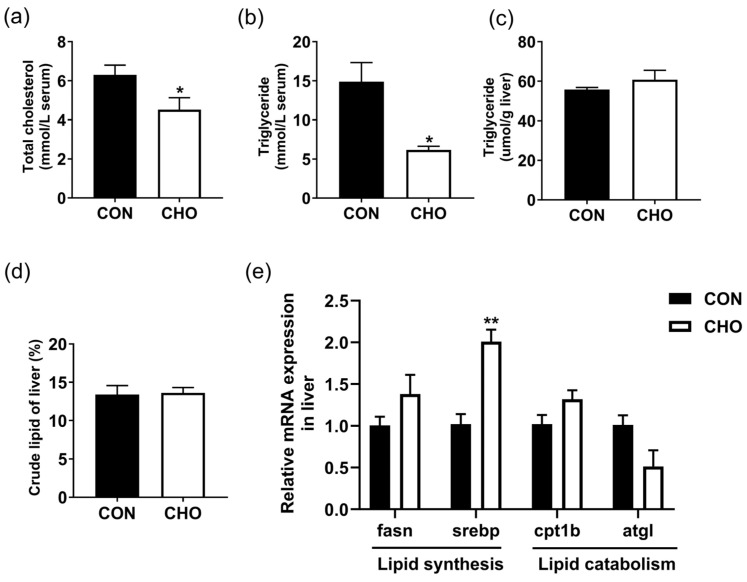
Effects of chronic hypoxia on lipid metabolism of turbot. (**a**) Serum total cholesterol content; (**b**) serum triglyceride content; (**c**) liver triglyceride content; (**d**) liver crude lipid content; (**e**) The expression of genes related to liver lipid metabolism. Note: CON: control normoxia group; CHO: chronic hypoxia group. “*” and “**” mean significant differences (*p* < 0.05), and (*p* < 0.01) respectively, between CON and CHO groups.

The serum total cholesterol and triglyceride contents were significantly reduced (Figure 7a,b), and the liver expression of *srebp* gene was significantly up-regulated in the CHO group than the CON group (Figure 7e). Meanwhile, no significant differences were observed in the liver triglyceride, crude lipid contents, and lipid catabolism-related genes (*cpt1b* and *atgl*) expression (Figure. 7c–e). These results suggest that chronic hypoxia could reduce serum triglyceride level, but not affect liver lipid level.

### 3.9. Effects of Chronic Hypoxia on Liver Fatty Acid Compositions

In Table 4, the C18:0, C24:1n-9, C20:2n-6, C20:3n-6, C22:5n-3, C22:6n-3, and DHA/EPA levels were significantly decreased, while the C20:1n-9 content was significantly increased in the CHO group compared with the CON group. These results certify that chronic hypoxia primarily decreased the n-3PUFA level in the liver.

## 4. Discussion

In aquaculture, high stocking density is the main cause of chronic hypoxia in aquatic animals. In nature, global warming decreases oxygen solubility and the rate of oxygen resupply from the atmosphere to the ocean. Meanwhile, eutrophication accelerates oxygen consumption by phytoplankton and microorganisms in coastal waters [5]. In North Carolina estuaries, chronic hypoxia (DO = 1.5 mg/L) has become a common occurrence during summer, leading to 31–89% growth reductions in Atlantic menhaden (*Brevoortia tyrannus*) and spot (*Leiostomus xanthurus*) [24]. In tiger puffer (*Takifugu rubripes*), chronic hypoxia (DO = 3.5 ± 0.5 mg/L) for 4 weeks significantly decreased the final body weight, weight gain, body indices, feed intake, and feed efficiency [2]. Similarly, chronic hypoxia has also been shown to reduced growth and feed intake in Nile tilapia [25] and European sea bass (*Dicentrarchus labrax*) [26]. In common carp (*Cyprinus carpio*), chronic hypoxia (DO = 10% oxygen saturation) for 8 days reduced feed intake by 79% via upregulating the expression of the potent anorexigenic gene leptin and its receptor [27]. Chronic hypoxia (DO = 40% oxygen saturation) also resulted in a significant reduction in protein digestibility, and an increase in digestible energy in feces of European sea bass (*Dicentrarchus labrax* L.) [28]. In juvenile cobia (*Rachycentron canadum*), chronic hypoxia (DO = 3.15± 0.21 mg/L) for 28 days significantly decreased the activities of digestive enzymes (trypsin, amylase, and lipase), intestinal muscle thickness, mucosal fold height, villus width, and the expression of tight junction protein-related genes (*zo1*, *zo2*, *occluding,* and *claudin4*) [29]. In Nile tilapia, dietary organic acids did not affect growth or nutrient digestibility under normoxia, but significantly enhanced growth and nutrient digestibility under hypoxia conditions (DO = 50% oxygen saturation) [30]. Hypoxia can trigger an elevation in ROS production from the mitochondrial respiratory chain, leading to a greater diversion of energy to counteract oxidative stress. In golden pompano (*Trachinotus blochii*), chronic hypoxia (DO = 3.0 ± 0.2 mg/L) for 2 weeks induced oxidative stress, inflammatory responses, and hepatic cell apoptosis [31]. In mirror carp (*Cyprinus carpio*), chronic hypoxia (DO = 1.8 ± 0.6 mg/L) for 21 days significantly enhanced the expression of *hif1α*, the DNA oxidative damage gene *ogg1*, base excision repair gene *xrcc1*, lipid peroxidation in the liver, and histopathological damage in the gill [32]. In this study, the weight gain, viscerosomatic index, condition factor, and oxygen consumption rate of turbot were significantly decreased, while the feed conversion ratio, the contents of the crude protein and crude lipid in the feces were significantly higher in the chronic hypoxia group than in the normoxia group. Collectively, these findings indicate that chronic hypoxia reduces appetite and feed intake, digestion and absorption of nutrients, and induces oxidative stress and damage, thus inhibiting fish growth.

Flesh quality is determined by a combination of multiple factors, such as muscular fiber, texture, connective tissue, flavor nucleotides, collagen, lactate, and pH levels, fatty acids, umami amino acid compositions, among others [33]. The muscles of most fish can grow continuously, and the newly formed myofibers are smaller, resulting in the coexistence of both small and large myofibers. Studies in fish have found that harder muscle is associated with higher protein levels, higher myofiber density, and smaller myofiber diameters [34,35]. In Atlantic salmon (*Salmo salar* L.), 12% reductions in muscle fiber number and cross-sectional fiber area were observed under hypoxia conditions (DO = 50% saturation) [36]. In Nile tilapia, chronic hypoxia (DO = 1.1 ± 0.1 mg/L) for 8 weeks decreased the muscle yield, protein, amino acid, glycogen, and n-3 PUFA contents, as well as pH, water-holding capacity, and the expression of collagens synthesis-related genes (*colia1/2*) [37]. In this study, the CHO group exhibited lower muscle hardness, springiness, and chewiness compared to the CON group. Correspondingly, chronic hypoxia decreased the muscle’s total amino acid, soluble protein, glycogen contents, and myofiber numbers, while increasing myofiber diameters, and muscle lactate content of turbot. In largemouth bass, chronic hypoxia reduced the expression of protein synthesis-related genes (*mtor* and *s6*), but up-regulated the expression of protein degradation-related genes (*foxo1a*, *foxo3a*, *beclin1*, *atg5*, *atg16l1*, *capns1a*, and *capns1b*), and increased SOD and glutathione peroxidase (GPX) activities in muscle [14]. In blunt snout bream (*Megalobrama amblycephala*), chronic hypoxia (DO = 2.0 mg/L) for 4 and 7 days significantly changed the morphological structure of the gill, increasing the lamellar respiratory surface area, TUNEL-positive apoptotic cell counts, the expression of pro-apoptotic gene (*bad*), and the activities of oxidative stress-related enzymes (SOD, CAT, and GSH) [38]. In goldfish (*Carassius auratus*), hypoxia (DO = 20% oxygen saturation) for 4 and 20 days increased the muscular protein levels of antioxidant enzyme (SOD), a regulator of the antioxidant cell response (Nrf2), and a transcription factor involved in mitochondrial dynamics (Pgc1α), indicating that oxidative balance and mitochondria play a crucial role in the process of skeletal muscle adaptation to hypoxia [39]. Based on the above findings, chronic hypoxia induces oxidative damage and apoptosis of muscle cells, and inhibits myofiber generation and development, thereby reducing numbers, density and hardness of myofiber. Oxidative stress can also induce lipid and protein peroxidation, leading to the breakdown of unsaturated fatty acids, changes in umami amino acids and flavor nucleotides, and an increase in unpleasant rancid flavors. Meanwhile, chronically elevated cortisol levels induced by hypoxia can promote protein catabolism, and the denaturation of myofibrillar proteins, contributing to reduced muscle hardness and altered texture. In addition, chronic hypoxia can lead to energy metabolic disturbances, including severe depletion of glycogen level, substantial accumulation of lactate, reduce the pH and flesh quality.

The Hif signaling pathway is the most important pathway for responding to hypoxia stress and reprogramming energy metabolism [40]. Hypoxia also acts directly on the insulin pathway and protein kinase B (Akt) [41]. The following studies have shown that hypoxia affected the three hifα isoforms expression, as well as glucose, lipid and protein metabolism in fish. In turbot, acute hypoxia (DO = 2.0 ± 0.5 mg/L) for 6–12 h promoted hemoglobin synthesis and anaerobic glycolysis (glycogen breakdown and lactate production) by activating *hif3α* in the liver and muscle, while inhibited lipid catabolism (*atgl, cpt1b*) and protein synthesis (*mtor*) in muscle [19]. In Nile tilapia, acute hypoxia (DO = 0.7 ± 0.1 mg/L) for 6 h significantly decreased liver and muscle glycogen content, up-regulated the anaerobic glycolysis pathway and expression of glycogenolysis-related genes, but did not alter the lipid metabolism. However, chronic hypoxia (DO = 1,1 ± 0.1 mg/L) for 4 weeks significantly reduced the lipid content and increased the liver glycogen content, fatty acid oxidation rate, and expression of lipolysis and carbohydrate synthesis-related genes in Nile tilapia [25]. The fish liver is rich in lipids, and polyunsaturated fatty acids are susceptible to oxidation under stress conditions. In rainbow trout, hypoxia (DO = 4.0 ± 0.5 mg/L) for 4 weeks elevated the n-6 PUFA level and delta-6-desaturase (fatty acid synthesis enzyme) activity, but decreased n-3 PUFA content [42]. Moreover, chronic hypoxia (DO = 2.5 ± 0.2 mg/L) for 2 weeks reduced intestinal short-chain fatty acids in the river prawn (*Macrobrachium nipponense*) [43]. Similarly, this outcome was observed in our study, where chronic hypoxia significantly decreased whole fish lipid content, total cholesterol and triglyceride levels in the serum, and polyunsaturated fatty acids (C20:2n-6, C20:3n-6, C22:5n-3, C22:6n-3, and DHA/EPA) levels in the liver compared to the CON group. In triploid rainbow trout, acute hypoxia (DO = 2–5 mg/L) for 10 min increased glucose, triglyceride, globulin levels, lactate dehydrogenase activity, and *hif2α* gene expression, but did not change *hif1α* gene expression in the liver [44]. In adult hybrid sturgeon (*Acipenser schrenckii*♂ × *Acipenser baerii*♀), acute hypoxia (DO = 1.1 ± 0.1 mg/L or 3.5 ± 0.2 mg/L) for 24 h increased the red blood cell count, hemoglobin, lactic acid, cortisol, glucose, total protein levels, and lactate dehydrogenase activity. Meanwhile, the gene expression of *hif1α, hif2α,* and *hif3α* in the gill significantly increased after 6 h of hypoxic exposure but returned to normoxic levels after 24 h [45]. In tiger puffer, 4 weeks of chronic hypoxia activated glycolysis by increasing the gene expression of *hif1α* and *hif2α* in the liver, and *hif3α* in the muscle, whereas 8 weeks chronic hypoxia inhibited glycolysis by reducing the gene expression of *hif1α*, *hif2α*, and *hif3α* in both the liver and muscle [2,46]. In this study, chronic hypoxia increased the hemoglobin content and hepatic angiogenesis by activating the *hif1α/vegfa* pathway. Meanwhile, chronic hypoxia led to the conversion of glycogen to lactate in the muscle, but *hif3α* gene expression was decreased in both muscle and liver. A similar phenomenon was found in Gulf killifish, where acute hypoxia (DO = 1.2 ± 0.2 mg/L) for 6–24 h significantly increased Hif1α protein levels in the brain, ovary, and skeletal muscle, but did not affect the mRNA levels of *hif1α, hif2α,* and *hif3α* [47]. These results indicate that the increase in Hif1α protein does not depend on the increase in *hif1α* mRNA, suggesting that Hif1α protein is post-translationally regulated during the initial response to hypoxia. These findings indicate that the expression patterns of the three *hifα* isoforms and change in energy metabolism are influenced by the duration and intensity of hypoxia, as well as by fish species and tissue types.

## 5. Conclusions

Dissolved oxygen is a critical environmental factor that affects the growth and flesh quality of cultured fish. In this study, chronic hypoxia significantly reduced the digestibility, feed efficiency, weight gain, and body indexes of turbot. Meanwhile, chronic hypoxia reduced muscle nutrient content and myofiber numbers, thereby decreasing muscle hardness and chewiness and impairing overall flesh quality. In addition, chronic hypoxia increased the hepatic angiogenesis by activating the *hif1α/vegfa* pathway, and decreased the lipid and n-3 PUFA levels. Collectively, these results demonstrate that chronic hypoxia represses growth performance and flesh quality in turbot and provides practical references for optimizing dissolved oxygen management and economic benefit in intensive turbot aquaculture. Furthermore, our findings systematically reveal the adaptation mechanisms of flatfish to chronic hypoxia from the aspects of growth, energy metabolism, nutritional composition, and flesh quality, which enrich the theoretical basis of hypoxia physiology in marine benthic fish.

## Figures and Tables

**Table 1:** Formulation and nutrients composition of the experimental diet (g/100 g).

Dietary Ingredient (%)	
Fishmeal	41.00
Soybean protein concentrate	25.00
Wheat meal	21.00
Fish oil	6.00
Soybean lecithin	1.50
Vitamin premix ^a^	0.40
Mineral premix ^b^	0.80
Choline chloride	0.50
Butylated hydroxytoluene	0.02
Dimethyl-beta-propiothetin	0.10
Ca(H_2_PO_4_)_2_	1.50
Vitamin C	0.50
Carboxymethyl cellulose	1.68
Total	100.00
Nutrients composition:	
Moisture (%)	9.31
Crude lipid (%)	10.62
Crude protein (%)	49.46
Ash (%)	8.65

^a^ Vitamin premix (mg/g premix): Inositol, 80 mg; Pantothenate, 6 mg; Nicotinic acid, 20 mg; Folic acid, 2 mg; Vitamin A, 3.2 mg; Vitamin D, 0.5 mg; Vitamin E, 12 mg; Thiamine, 2.5 mg; Riboflavin, 4.5 mg; Vitamin B6, 2 mg; Vitamin B12, 0.01 mg; Biotin, 0.12 mg; Menadione, 1 mg; Wheat middling, 866.17 mg. ^b^ Mineral premix (mg/g premix): MgSO_4_·H_2_O, 50 mg; ZnSO_4_·H_2_O, 45.2 mg; MnSO_4_·H_2_O, 9.3 mg; CuSO_4_·5H_2_O, 3.7 mg; FeSO_4_·H_2_O, 112.7 mg; NaCl, 100 mg; CoCl_2_·6H_2_O, 0.4 mg; Ca(IO_3_)_2_, 0.3 mg; NaSeSO_3_, 0.1 mg; zoelite 678.3 mg.

**Table 2:** The qPCR primers.

Gene Name	Sequences (5′ to 3′) Forward and Reverse	Product Length	GenBank No.
*gck*	CTTCCGTGACAGGTTCCACA	117	XM_035639201.1
(glucokinase)	CAGGCCACCGCTGAGATAAG		
*pklr*	CAGTCGCAATGATGCACTCG	70	XM_035623804.2
(pyruvate kinase L/R)	TCCTCAAAGAGCTGCTGGTG		
*ldha*	TCCAAGGTGGTGGTGGTTAC	129	XM_035643663.1
(lactate dehydrogenase A4)	GCAGTTGGGGCTGTACTTGA		
*hif1α*	GACTACTACCGGGCACAAGG	82	XM_035610073.1
(hypoxia-inducible factor 1 subunit alpha a)	CTCAATGTTGGAGGGGTGCT		
*hif2α/epas1b*	AGCAGACGGAGACCTTGTTCA	185	XM_035605652.1
(endothelial PAS domain protein 1b)	ATCCGACAAACCGAAGTCGAGG		
*hif3α/hif1αl*	ATGCATGTTCGAAACCCAGC	136	XM_035623708.1
(hypoxia-inducible factor 1 subunit alpha, like)	ACGAACGACAGCTCGACAAA		
*vegfab*	CGACAAAACAATTCACGACGC	82	XM_035617034.1
(vascular endothelial growth factor Ab)	GTCTTGCACGAACAAACGCT		
*fasn*	CAACTCGTTCGGATTTGGCG	109	XM_035612875.1
(fatty acid synthase)	GGCATTCAGAACCCTGGGAA		
*srebf1*	GCTTCCAATCAACCGCATCG	163	XM_035615397.1
(sterol regulatory element binding transcription factor 1)	GAGCTTGGCCTCAGTACCAG		
*atgl/pnpla2*	ATGTCGCGAAAGAGGCAAGA	85	XM_035643755.1
(patatin-like phospholipase domain containing 2)	CGCAGCATATGACGCACAAT		
*cpt1b*	GTATGTTCCGAGACGGACGG	87	XM_035642881.1
(carnitine palmitoyltransferase 1B)	CACTTGTGTCCTCCATGGCT		
*mtor*	GGTTGGGAGCAGACAGGAAT	94	XM_035644866.1
(mechanistic target of rapamycin kinase)	TGCTGGAAGAAGAACGTGGG		
*glut2l*	TACTGCGGGTTGACATCTGG	149	XM_035609382.1
(solute carrier family 2 member 2)	CCAAGCACAAAGTCCAAGCC		
*gcn2/eif2ak4*	TCAGCCCAGAAAAGGTGTCC	125	XM_035609728.1
(eukaryotic translation initiation factor 2 alpha kinase 4)	GTCCACCGCCAGAATCTCAA		
*uba1*	CATCCGCCAGCTACTTCACA	99	XM_035644760.1
(ubiquitin-like modifier activating enzyme 1)	AATTCTAGGGGGTGGGGACA		
*ir*	TTACGTCAGTGCCCGAACAA	84	XM_035647756.1
(insulin receptor b)	GCCTCAGCCGTCACTATCTC		
*hk1*	GATGACGGACGGATGTGTGT	160	XM_035628212.1
(hexokinase-1)	CACCAAGGTACATGCCGCTA		
*irs2*	CGGCAGCAACAACAACAACATC	142	XM_035603815.1
(insulin receptor substrate 2b)	CCATTTCTTCTCGCTCTCGTAG		
*glud1*	TCCCCATCAAGAGAGACGGC	165	XM_035605065.1
(glutamate dehydrogenase 1a)	CCACAGCACACTTGTAGGTCA		
*gsk3β*	GTCAGCAGGGATAAGGATGG	100	XM_035651470.1
(glycogen synthase kinase 3 beta, genome duplicate a)	CCTTAGTGTCCGTGTAGCTC		
*akt1*	TCTTCGCGGGAATCGAATGG	99	XM_035609811.1
(v-akt murine thymoma viral oncogene homolog 1)	CGTCAAAATACCGCGTGTCC		
*foxo3b*	TAGCGGGCTAGTCTCACCTT	113	XM_035618500.2
(forkhead box O3b)	AGGAACTTGTTGTGTGGGGG		
*pten*	CTGGACTTTTACGGCGAGGT	246	XM_035612234.2
(phosphatase and tensin homolog B)	CCGTGTGTGGGAGGAGTTAG		
*rpl19*	TGGATCCCAACGAGACCAAC	97	XM_035614206.1
(ribosomal protein L19)	GTGACAGGCTTGCGAATGAT		
*β-actin*	CTTCCCTTCTATCGTCGGTC	91	XM_035614479.1
(actin beta 2)	GCTCTGGGCTTCATCACCTA		

**Table 3:** Amino acid compositions in the muscle.

Amino Acid	CON	CHO
Threonine	3.52 ± 0.06	3.44 ± 0.09
Valine	4.16 ± 0.06	4.04 ± 0.10
Methionine	2.48 ± 0.06	2.37 ± 0.06
Isoleucine	3.79 ± 0.06	3.73 ± 0.08
Leucine	6.28 ± 0.09	6.11 ± 0.15
Phenylalanine	3.39 ± 0.04	3.27 ± 0.06
Lysine	7.14 ± 0.09	6.89 ± 0.19
Histidine	1.65 ± 0.02	1.63 ± 0.05
Arginine	4.95 ± 0.08	4.82 ± 0.12
∑EAA	37.35 ± 0.50	36.23 ± 0.88
Taurine	1.91 ± 0.06	1.78 ± 0.18
Aspartic acid	8.05 ± 0.15	8.12 ± 0.13
Serine	3.14 ± 0.05	3.08 ± 0.02
Glutamic acid	13.03 ± 0.25	13.00 ± 0.19
Glycine	3.44 ± 0.10	3.50 ± 0.08
Alanine	4.63 ± 0.07	4.52 ± 0.12
Cystine	0.98 ± 0.05	0.77 ± 0.07
Tyrosine	2.84 ± 0.04	2.75 ± 0.06
Proline	2.58 ± 0.04	2.58 ± 0.07
∑NEAA	41.29 ± 0.46	41.05 ± 0.33

Note: AA: amino acid; EAA: essential amino acid; NEAA, non-essential amino acid; ∑EAA: total essential amino acids; ∑NEAA: total non-essential amino acids; CON: control normoxia group; CHO: chronic hypoxia group.

**Table 4:** Fatty acid compositions in the liver.

Fatty Acid	CON	CHO
C14:0	4.33 ± 0.09	4.47 ± 0.06
C15:0	0.44 ± 0.03	0.51 ± 0.03
C16:0	14.84 ± 0.18	14.69 ± 0.27
C17:0	0.52 ± 0.03	0.46 ± 0.04
C18:0	3.06 ± 0.17	2.47 ± 0.13 *
C20:0	0.21 ± 0.02	0.16 ± 0.02
∑SFA	23.33 ± 0.27	22.58 ± 0.27
C14:1n-5	0.06 ± 0.01	0.17 ± 0.07
C16:1n-7	14.84 ± 0.18	14.69 ± 0.27
C17:1n-7	0.31 ± 0.04	0.39 ± 0.04
C18:1n-9	26.45 ± 0.62	23.91 ± 0.86
C20:1n-9	2.36 ± 0.10	3.19 ± 0.24 *
C22:1n-9	0.54 ± 0.05	0.67 ± 0.10
C24:1n-9	0.17 ± 0.02	0.06 ± 0.01 **
∑MUFA	35.67 ± 1.27	34.54 ± 1.08
C18:2n-6	16.86 ± 0.8	18.11 ± 0.17
C18:3n-6	0.28 ± 0.01	0.28 ± 0.02
C20:2n-6	2.12 ± 0.04	1.57 ± 0.07 ***
C20:3n-6	0.52 ± 0.03	0.05 ± 0.01 ***
C22:2n-6	0.06 ± 0.01	0.07 ± 0.01
∑n-6PUFA	19.1 ± 0.59	20.47 ± 0.13
C20:3n-3	0.74 ± 0.04	0.62 ± 0.08
C20:5n-3	6.36 ± 0.46	6.81 ± 0.24
C22:5n-3	3.90 ± 0.07	3.22 ± 0.19 *
C22:6n-3	12.14 ± 0.24	10.44 ± 0.37 **
∑n-3PUFA	23.02 ± 0.54	21.1 ± 0.50 *
DHA/EPA	1.93 ± 0.09	1.60 ± 0.05 **

Note: SFA: saturated fatty acids; MUFA, monounsaturated fatty acids; PUFA: polyunsaturated fatty acids; ∑SFA: total saturated fatty acids; ∑MUFA: total unsaturated mono-fatty acid: ∑PUFA: total polyunsaturated fatty acid; CON: control normoxia group; CHO: chronic hypoxia group; C20:5n-3: eicosapentaenoic acid (EPA); C22:6n-3: docosahexaenoic acid (DHA). “*”, “**”, and “***” mean significant differences (*p* < 0.05), (*p* < 0.01), and (*p* < 0.001) respectively, between CON and CHO groups.

## Data Availability

All Data were available from the corresponding author on reasonable request.
